# Fatal Case of Legionnaires’ Disease After Home Exposure to *Legionella pneumophila* Serogroup 3 — Wisconsin, 2018

**DOI:** 10.15585/mmwr.mm6908a2

**Published:** 2020-02-28

**Authors:** Amy Schumacher, Anna Kocharian, Amanda Koch, John Marx

**Affiliations:** ^1^Epidemic Intelligence Service, CDC; ^2^Bureau of Communicable Diseases, Division of Public Health, Wisconsin Department of Health Services; ^3^Infection Control, UW Health, Madison, Wisconsin.

In January 2018, the Wisconsin Department of Health Services, Division of Public Health (DPH), received a report of a culture-confirmed case of Legionnaires’ disease. The patient, who was immunocompromised, had died at a local hospital 10 days after being admitted. DPH and an infection preventionist from the hospital investigated to determine the source of the infection and prevent additional cases. Because the case was suspected to be nosocomial, health care facility water samples were tested for *Legionella*. When these samples were negative, water sources in the patient’s home were tested. These tested positive for *Legionella pneumophila,* and the bacteria remained after an attempt to remediate. The patient and home isolates were identified as *L. pneumophila* serogroup 3, sequence type 93, by whole-genome multilocus sequence typing. A second resident of the home did not become ill. This case highlights the potential for immunocompromised persons and others at risk for Legionnaires’ disease to be exposed to *Legionella* through home water systems containing the bacteria and demonstrates the difficulty of home remediation. This case also illustrates the role of lower respiratory tract specimens in the identification of less common *Legionella* infections (e.g., *L. pneumophila* serogroup 3) and confirmation of the infection source.

## Investigation and Results

The case occurred in a Wisconsin resident, a nonsmoker aged 70–79 years who had received a 2016 diagnosis of late-onset combined immunodeficiency of undetermined etiology after developing two opportunistic infections that year. The patient was admitted to a local hospital (day 0) (Figure) after evaluation in the emergency department for a rash and fever suspected to be a reaction to oral antibiotics. The patient had been prescribed a 30-day course of oral antibiotics (levofloxacin) following a course of parenteral antibiotics for management of cellulitis of the lower leg with accompanying abscesses. The rash and fever developed near the end of the course of oral antibiotics, which was stopped a day early. On admission, a family member reported recent mental status changes in the patient, and the patient complained of a dry cough and shortness of breath. A respiratory virus panel on sputum detected rhinovirus and parainfluenza 1, but a chest radiograph was normal.

**FIGURE:**
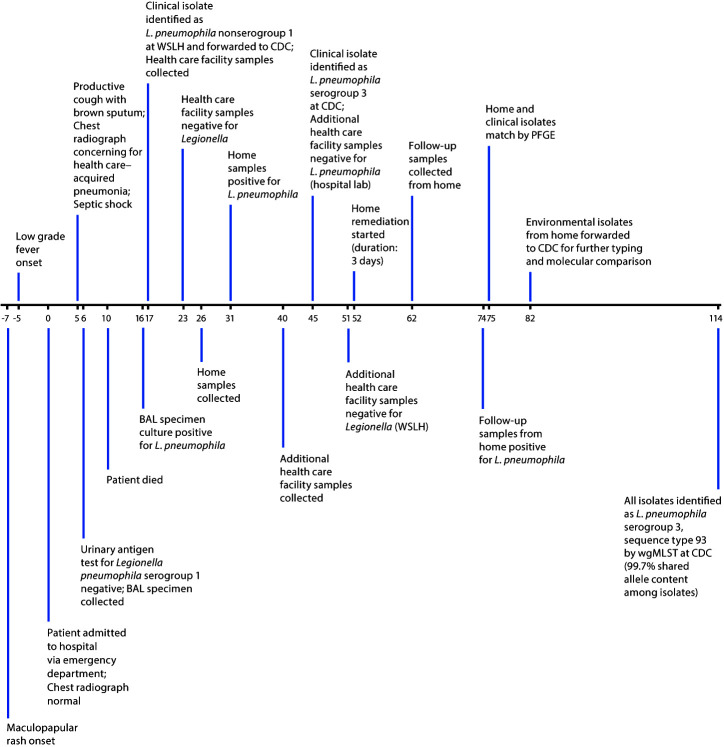
Timeline of events during investigation of a fatal case of Legionnaires’ disease — Wisconsin, 2018* **Abbreviations:** BAL = bronchoalveolar lavage; PFGE = pulsed-field gel electrophoresis; wgMLST = whole-genome multilocus sequence typing; WSLH = Wisconsin State Laboratory of Hygiene. *Approximate days before and recorded days after patient’s hospital admission (day 0).

After admission, the fever continued, and mental status changes worsened. The cough became productive with dark brown sputum on day 5; a chest radiograph showed a new right upper lobe opacity. The patient developed septic shock, thought to be attributable to hospital-acquired pneumonia, and was transferred to the intensive care unit. Intravenous aztreonam and vancomycin were initiated, with aztreonam replaced by meropenem later in the day. On day 6, a urinary antigen test for *L. pneumophila* serogroup 1 and culture of a bronchoalveolar lavage (BAL) fluid specimen for *Legionella* were ordered; the urinary antigen test result was negative. Inhaled tobramycin was initiated on day 7. Vancomycin and tobramycin were discontinued on day 8. The patient died on day 10, noted in the medical record as the result of cardiopulmonary arrest secondary to septic shock. On day 16, the Wisconsin State Laboratory of Hygiene (WSLH) isolated *L. pneumophila* from the BAL fluid culture. The isolate was identified on day 17 as *L. pneumophila* nonserogroup 1 by direct fluorescent antibody and determined later in the investigation to be serogroup 3, which is not detected by the urinary antigen test. Serogroup 3 is not often reported as a cause of Legionnaires’; serogroup 1 accounts for most infections (*1*).

The hospital’s water management program requires certain actions to be taken in the event that any patient tests positive for *Legionella* by culture or urinary antigen test with symptom onset indicating possible nosocomial acquisition (i.e., inpatient for more than 2 days or discharged from an inpatient location within 10 days). The program was instituted as a result of a Legionnaires’ disease outbreak during the 1990s and involves targeted environmental cultures. Whereas targeted sampling is typical, this hospital also had the capacity to culture for *Legionella* in-house. Nine water sources (inpatient and outpatient sink faucets, ice machines, a shower, and a warm water pool) where the patient might have been exposed were sampled (day 17) (Table). Samples were cultured for *Legionella* at the hospital; all culture results were negative. Additional samples were collected on day 40 and cultured for *Legionella* at both the hospital and WSLH for validation. All culture results were negative.

**TABLE:** Environmental sampling and culture results from a health care facility and a personal residence during investigation of a fatal case of Legionnaires’ disease — Wisconsin, 2018

Sampling site	Days after patient’s hospital admission	Sample type*	Aerator/ Showerhead removed^†^	Affiliation of laboratory	*Legionella pneumophila* culture result	CFU ml
**Health care facility**						
**Outpatient clinic**						
Exam room faucet	17	Bulk (550 ml), initial	No	Hospital	Neg	—
Warm water pool	17	Bulk (550 ml)	N/A	Hospital	Neg	—
**Emergency department**						
Exam room faucet	17	Bulk (550 ml), initial	No	Hospital	Neg	—
Ice machine	17	Bulk (550 ml)	N/A	Hospital	Neg	—
**Inpatient**						
Patient room sink faucet	17	Bulk (550 ml), initial	No	Hospital	Neg	—
		Bulk (550 ml), 2 min	No	Hospital	Neg	—
	40	Bulk (550 ml), warm	No	Hospital	Neg	—
				WSLH	Neg	—
		Swab	Yes	Hospital	Neg	—
				WSLH	Neg	—
Patient room shower	17	Bulk (550 ml), initial	No	Hospital	Neg	—
		Bulk (550 ml), 2 min	No	Hospital	Neg	—
	40	Bulk (550 ml), warm	No	Hospital	Neg	—
				WSLH	Neg	—
		Swab	Yes	Hospital	Neg	—
				WSLH	Neg	—
Kitchen sink	17	Bulk (550 ml), initial	No	Hospital	Neg	—
Ice machine	17	Bulk (550 ml)	N/A	Hospital	Neg	—
**Intensive care unit**						
Patient room sink	17	Bulk (550 ml), initial	No	Hospital	Neg	—
	40	Bulk (550 ml), warm	No	Hospital	Neg	—
				WSLH	Neg	—
		Swab	Yes	Hospital	Neg	—
				WSLH	Neg	—
**Patient home samples**						
**Bathroom 1**						
Shower	26	Bulk, 550 ml, warm	No	Hospital	Pos	2.54
	62	Bulk (250 ml), warm	Yes	WSLH	Neg	—
		Swab	Yes	WSLH	Neg	—
**Bathroom 2**						
Shower	26	Bulk, 550 ml, warm	No	Hospital	Pos	0.36
	62	Bulk (250 ml), warm	Yes	WSLH	Neg	—
		Swab	Yes	WSLH	Neg	—
Shower wand	62	Bulk (250 ml), warm	No	WSLH	Neg	—
Sink	62	Bulk (250 ml), warm	No	WSLH	Pos	0.05
**Kitchen**						
Sink #1 sprayer	62	Bulk (250 ml), warm	No	WSLH	Pos	0.11
Sink #2 sprayer	62	Bulk (250 ml), warm	No	WSLH	Pos	0.26
**Entire home**						
Water heater	62	Bulk (250 ml)	N/A	WSLH	Neg	—
Humidifier	62	Swab	N/A	WSLH	Neg	—

**Abbreviations:** CFU = colony forming units; N/A = not applicable; Neg = no *Legionella pneumophila* isolated; Pos = *Legionella* isolated; WSLH = Wisconsin State Laboratory of Hygiene.

* Bulk samples were either drawn immediately when the faucet was turned on, after 2 minutes of hot water flow, or after running the water until warm.

^†^ Sinks have aerators, and showers have showerheads. Removal of the aerator or showerhead is required to obtain a swab sample. Procedural differences or inability to remove an aerator or showerhead for a fixture caused the variation.

On day 26, and at the request of the patient’s family, staff members from the hospital collected and cultured samples from two showers in the patient’s residence, a single-family home built in the early 1910s that appeared to be in good condition and was served by a municipal water supply system. Both cultures were positive for *L. pneumophila*. Starting on day 52 and following advice in a 2010 home guidance manual (*2*), a plumber drained, cleaned with chlorine, and superheated to 148°F (64.4°C) the home’s water heater and then flushed the fixtures over a 3-day period. The showerheads were also soaked in vinegar to remove hard water scale. As part of the case investigation and to determine whether remediation was successful, DPH epidemiologists collected samples in the home on day 62. Eight fixtures were sampled: the two showers, one handheld shower wand, a bathroom sink faucet, two kitchen sink sprayers, the water heater tank, and the whole-house humidifier. Samples were cultured for *Legionella* at WSLH. Cultures from the bathroom faucet and the kitchen sprayers were positive for *L. pneumophila*, indicating that the bacteria remained in the home’s water system. The home guidance manual (*2*) was shared with the other resident of the home, who was not immunocompromised and did not become ill. Practices to prevent *Legionella* growth within the water system were discussed, such as adhering to recommended water heater settings and maintenance schedules and flushing fixtures after lack of use (e.g., after returning from vacation). The other resident also was encouraged to seek medical advice if they developed symptoms of respiratory illness.

On day 75, WSLH reported that the isolates from the home environmental samples collected on day 62 and the patient’s clinical isolate matched by pulsed-field gel electrophoresis patterns. The isolates were sent to CDC for whole-genome multilocus sequence typing and on day 114 were identified as *L. pneumophila* serogroup 3, sequence type 93, with 99.7% shared allele content among strains. The patient’s home was determined to be the most likely source of infection.

## Discussion

In Wisconsin, the number of reported confirmed Legionnaires’ disease cases has increased more than 400% from 63 in 2010 to 330 in 2018; this increase is consistent with a national 4.5-fold increase in the incidence rate from 2000 to 2015 (*1*). Approximately 9% of Legionnaires’ disease cases in the United States are fatal (*3*), and two thirds of patients with confirmed cases during 2015 had not traveled or been in a health care or assisted living setting during their presumed exposure period (*1*).

This Wisconsin case highlights the potential for persons at risk for Legionnaires’ disease (e.g., patients with a weakened immune system) to be exposed to *Legionella* through home water systems containing the bacteria and also demonstrates the difficulty of remediation. Evidence of *Legionella* in residential potable water has been widely documented (*4*). Published guidance for prevention of *Legionella* growth and spread is limited to complex building water systems through water management programs and, when indicated, immediate control measures such as point-of-use filters (*5*,*6*). Evidence regarding the burden (i.e., morbidity and mortality) from home-associated Legionnaires’ disease and the usefulness and feasibility of testing and remediation in residential settings is limited. Increased efforts to understand the burden and risk associated with residential settings could inform prevention guidance.

This case also highlights the value of obtaining lower respiratory tract specimens for both patient treatment (identification of less common *Legionella* infections) and public health investigation (confirmation of infection source). Nonserogroup 1 *L. pneumophila* infections cannot be routinely detected using the *Legionella* urinary antigen test but can be detected by polymerase chain reaction or culture of lower respiratory tract secretions (e.g., sputum or BAL fluid). Importantly, sputum specimens can be successfully cultured for *Legionella* even if the specimen is considered low quality (*7*). Because timely antibiotic treatment can affect patient survival, CDC recommends concurrent ordering of *Legionella* urinary antigen test and a culture of lower respiratory tract secretions for patients with suspected Legionnaires’ disease (*8*).

**Summary**

**What is already known about this topic?**

Legionnaires’ disease is a severe pneumonia caused by the waterborne bacteria *Legionella*. The rate of reported cases in the United States increased 4.5-fold from 2000 to 2015.

**What is added by this report?**

Investigation of a culture-confirmed fatal case of Legionnaires’ disease reported in January 2018 identified the patient’s home as the infection source. *Legionella* bacteria remained following a remediation attempt.

**What are the implications for public health practice?**

Increased efforts to understand the burden (i.e., morbidity and mortality) and risk for Legionnaires’ disease associated with residential settings and the usefulness and feasibility of testing and remediation in these settings could inform prevention guidance. Lower respiratory tract specimens have value for both patient treatment and public health investigation.
